# The role of physiotherapy in the treatment of HIV-related sensory neuropathy: The perceptions and referral practices of physicians

**DOI:** 10.4102/sajp.v71i1.286

**Published:** 2015-11-27

**Authors:** Tania Steyl, Felista T. Shayo

**Affiliations:** 1 Department of Physiotherapy, University of the Western Cape, South Africa

## Abstract

**Background:**

HIV-related peripheral neuropathies are among the most prevalent chronic neurological disorders affecting persons living with HIV and AIDS. In order to improve the physical function and quality of life of those affected by the disease, a holistic or multidisciplinary approach, including physiotherapy, has been suggested for the management of neuropathic pain.

**Aim:**

The aim of this study was to explore the physicians’ perceptions regarding the role of physiotherapy in the management of patients with HIV-sensory neuropathy (HIV-SN) and their referral practices in Tanzania.

**Methods:**

A qualitative study design incorporating purposive sampling was employed in the study. A total of 10 physicians from a hospital in Tanzania agreed to participate in in-depth interviews.

**Results:**

Physicians had poor perceptions of the role of physiotherapy in the management of patients with HIV-SN. Their inadequate knowledge of the role of physiotherapy and the limited number of physiotherapists employed negatively influenced their referral of patients with HIV-SN for physiotherapy.

**Conclusion:**

In Tanzania, referral for physiotherapy is still dependent on medical doctors. Inter-professional learning is imperative for minimising the stereotypes that may exist across professions, hence the need to improve awareness of specific roles in patient management. This could improve knowledge of the role of other professionals in the management and rehabilitation of affected patients and consequently improve perceptions and facilitate referrals of patients with HIV-SN for more integrated care.

## Introduction

It has been well established that the human immunodeficiency virus and Acquired Immune Deficiency Syndrome (HIV and AIDS) epidemic has reached alarming proportions globally (World Health Organization [WHO] 2012). The Joint United Nations Programme on HIV and AIDS reported that at the end of 2011 a total of 34 million people were living with HIV and AIDS, with sub-Saharan Africa accounting for 23.5 million (69%) of all the people living with HIV and AIDS worldwide. At the end of 2011, the number of people living with HIV and AIDS in Tanzania was estimated to be 1.5 million of the general population of 44.9 million people (WHO 2012). The HIV and AIDS epidemic in Tanzania has a clear impact on all sectors of development through the debilitation and depletion of the economically active population, especially young people. The disease has affected the most economically active group ranging from 15–45 years (Tanzania Commission for AIDS [TACAID] 2012).

Since the introduction of highly active antiretroviral treatment (HAART) nearly two decades ago, HIV has dramatically changed from being a fatal illness to a chronic condition with long-term management of symptoms, maintaining the independence and improving the quality of life of those affected (Hillier *et al.* 2010). Several studies have reported that HIV-related peripheral neuropathies are among the most prevalent chronic neurological disorders affecting persons living with HIV and AIDS (Phillips *et al.* 2010; Verma, Estanisloa & Simpson 2005; Wolfort & Dellon 2012). It is the most frequent neurological complication of HIV and AIDS in developed countries (Robinson-Papp & Sympson 2009) and in sub-Saharan Africa (Shurie & Deribew 2010). HIV-related peripheral neuropathies are classified into six different types, depending on the symptoms and the time of onset (Konchalard & Wangphonpattanasiri 2007). Of these, distal sensory neuropathy (HIV-SN) is the most common type, affecting more than one-third of the people living with HIV and AIDS (Biraguma & Rhoda 2012; Evans *et al.* 2011; Ghosh, Chandran & Jansen 2012). The clinical manifestation of HIV-SN presents as symmetrical symptoms of pain (burning or shooting), numbness and paresthesia (pins and needles), hyperaesthesia (increased contact sensitivity) and weakness of the muscles in the feet and legs and sometimes in the hands and arms, leading to functional limitation and disturbance of sleep****** (Markarian, Wulff & Simpson 2012; Verma *et al.* 2005).

People living with HIV-SN are more at risk of being unemployed, dependent in their activities of daily life (ADL) and more likely to have major depressive disorders (Ellis *et al.* 2010). It is therefore important to address the conditions that significantly impair their quality of life (Gale 2003; Verma *et al.* 2005). The management of HIV-SN is a major clinical challenge of global significance (Cherry *et al.* 2009). The available treatment is limited to symptomatic measures with limited efficacy (Evans *et al.* 2011). Medical management alone is inadequate and is unable to control the devastating pain of HIV-SN (Chetty *et al.* 2012; Gale 2003; Verma *et al.* 2005). Hence, a holistic or multidisciplinary approach, including physiotherapy, has been suggested for the management of neuropathic pain in order to improve physical function and quality of life for those affected by the disease (Chetty & Maharaj 2013; Gilron *et al.* 2006; Uwimana 2005). It is evident that physiotherapy management can play an integral role in the treatment of people with HIV-SN in order to improve their quality of life (Gale 2003; Gilron *et al.* 2006; O’ Brien & Nixon 2010).

In most African countries, physicians are the primary health practitioners responsible for the referral of patients to other health practitioners, including physiotherapists (Odebiyi *et al.* 2010). Tanzania is a country where a referral from a physician is needed in order to obtain physiotherapy treatment. Furthermore, the referral of patients for physiotherapy depends on the perceptions and the awareness of the referring physician regarding the role that physiotherapy can play in alleviating the condition of the patient (Quartey *et al.* 2009). Research also reported a deficit in physicians’ awareness of the role of physiotherapy in healthcare delivery (Odebiyi *et al.* 2010; Stanton *et al*. 1985), and specifically in the management of patients with HIV-SN (Chetty *et al.* 2012). In addition, Worthington *et al.* (2005) noted that negative perceptions of physicians regarding the role of rehabilitation members of the multidisciplinary team, including the physiotherapists, contributed to a lack of referral for the more integrated management of people living with HIV and AIDS in Australia. Therefore the aim of this study was to explore the physicians’ perceptions regarding the role of physiotherapy in the management of patients with HIV-SN and their referral practices in Tanzania.

## Methods

### Research setting

The study was conducted in the United Republic of Tanzania, the largest country in the eastern part of Africa with a general population of 44.9 million people (National Bureau of Statistics 2013). The data were collected at a tertiary referral hospital in Dar es Salaam catering for patients referred from both private and public hospitals in the Dar es Salaam region and from all other regions of the country. The hospital also hosts the busiest HIV care and treatment clinic in the country (Minzi & Naazneen 2008).

### Study design and participants

A qualitative study design was employed in this study. The study incorporated purposive sampling. Therefore all 15 physicians working at the HIV clinic of the conveniently selected hospital at the time of data collection were invited to participate in the in-depth interviews. A total of 10 physicians agreed to participate in the study.

### Procedure

Ethical considerations were taken into account before embarking on this study. Permission to conduct the study was obtained from the UWC Higher Degrees Committee as well as the Medical Director of the selected hospital. Physicians were approached by the researcher after their usual clinical meeting in the conference room at the hospital. Written informed consent was obtained from 10 of the physicians willing to participate in the study prior to the face-to-face interviews. Participants were asked to reflect on their opinion regarding the role of physiotherapy in the management of HIV-related sensory neuropathy. One open-ended question with probes were used and participants were encouraged to talk freely and participate fully in the audiotaped interviews conducted by the researcher in English. A trained research assistant took field notes.

### Data analysis

Data from the audiotape recordings were transcribed verbatim by an independent person with experience in transcription in order to produce findings and results. A comparison was made between notes taken during the discussions as well as with the audiotape recordings to verify accuracy. The authors read through the transcripts several times. Thereafter content analysis was done by extracting meaningful ideas of the participants’ opinions (coding into themes). Grouping the themes into broader categories was done in order in order to fit small categories together. After the derivation of themes, an independent researcher read through the transcripts and generated themes that were then compared to the themes of the researcher.

The trustworthiness of qualitative data is measured by its credibility, which in qualitative research is determined by the match between the constructed reality of the participants and the reality presented by the researcher (Lincoln & Guba 1985). Several steps were considered to build credibility: prolonged engagement and persistent observation; member checks by giving feedback of the data to the participants so that they could comment on the accuracy of the transcripts; responses were transcribed verbatim and independent researchers were asked to read through the transcripts and generate the themes.

## Results

A total of 10 physicians agreed to participate in the study. The mean age of the group was 44.9 years (s.d. = 34.766); three were females and seven males; years of working experience ranged between two and 13 years. The content analysis yielded various themes, as outlined below.

The participants expressed a wide range of perceptions about the role of physiotherapy in the management of patients with HIV-related sensory neuropathy (HIV-SN). The majority of the participants acknowledged that their perception is one of the factors that influenced their practice of referring the patients diagnosed with HIV-SN for physiotherapy management.

### Physiotherapists’ role

The majority of the physicians felt that physiotherapists have no significant role to play in the management of patients with HIV-SN, as demonstrated below:

‘I don’t think there is a significant role of physiotherapy in patients with HIV-related sensory neuropathy, because as for me medical treatment is the most important for these patients.’ (Participant 1, Female, 40 years)

‘I think there is no role of physiotherapy in these cases. If the patient has some functional limitation, I always ask the relatives to perform some exercises at home.’ (Participant 2, Male, 45 years)

‘Physiotherapists are not responsible for the management of these patients; these are medical cases and I do not expect much from a physiotherapist. So I would rather make a follow up with medication.’ (Participant 3, Male, 41 years)

However, some physicians indicated that physiotherapists have various roles to play in the care of patients with HIV-SN, including joint and muscle mobilisation as well as gait re-education. As two of them described:

‘Yeah, I think physiotherapists have a role to play. They should mobilise the patients to prevent muscle atrophy and functional limitation.’ (Participant 4, Male, 59 years)

‘There is a big role to play, because these patients sometimes have difficulties with walking. I think physiotherapists should take the responsibility of gait training so that the patient can be able to walk properly.’ (Participant 5, Male, 43 years)

### Referral practices of physicians

The referral practices of physicians differ a lot. The physicians either rarely refer or do not refer patients with HIV-SN at all for physiotherapy management. This is reflected in the statements below:

‘Very rare … do refer, but only for severe cases like the patients in wheel chairs.’ (Participant 6, Female, 39 years)

‘I recommend physiotherapy for refractory cases, but I do not refer every patient with HIV-related sensory neuropathy to physiotherapy. I usually refer them in advanced stage of the disease.’ (Participant 4, Male, 59 years)

‘It is very uncommon for me because the physiotherapists are very few compared to the number of patients here at the hospital and if you send the patient there, it always takes more than a month to get the first visit … and follow up for them is very poor and the patients do not get good results.’ (Participant 7, Female, 44 years)

‘I have never referred any patient with HIV-related sensory neuropathy to the physiotherapist. I think physiotherapists are more committed in the management of contractures and stiffness of the joints and peripheral neuropathy is a medical case.’ (Participant 6, Female, 39 years)

### Barriers to referral for physiotherapy management

Several barriers to referral were identified and are outlined below. Statements of physicians are included.

#### Physician’s knowledge

‘I do not understand the role of physiotherapy in the management of patients with HIV-related neuropathy. If I knew that my patients will benefit from physiotherapy, I would have referred them.’ (Participant 8, Male, 50 years)

‘Physiotherapists have not done much to tell the other professionals about what they do and which cases should be sent to a physiotherapist.’ (Participant 9, Male, 46 years)

‘I do not send patients with HIV-neuropathy for physiotherapy, as I do not understand their role in these patients. Honestly, physiotherapists have not made an effort to raise the awareness of what they are doing.’ (Participant 10, Male, 42 years)

#### Perceptions around patients’ awareness and attitudes

Some of the physicians claimed that patients’ lack of knowledge and awareness of the benefits of physiotherapy in the management of their disease made referral very difficult. The excerpts below demonstrate this view:

‘I would like to refer patients to physiotherapy, but it takes time to explain to them about the benefit of physiotherapy so as to convince them to attend. Otherwise they think of physiotherapy as simple exercises which they can do with the assistance of their relative at home.’ (Participant 9, Male, 46 years)

‘Patients think that they will be hurt by the exercises if they go to physiotherapists, especially the patients who have never been to physiotherapy previously.’ (Participant 3, Male, 41 years)

‘Patients don’t believe that physiotherapy can help alleviate their symptoms. They believe in drug treatment so it takes a lot of explanation to get the patient willing to attend physiotherapy.’ (Participant 1, Female, 40 years)

#### Not enough physiotherapists

The respondents expressed their concern at the limited number of physiotherapy staff members at the hospital:

‘There are only a few physiotherapists compared to the number of patients here at the hospital. For patients to get an appointment for the first time can take more than a month and follow up is very poor. Therefore I think the patients do not benefit much.’ (Participant 4, Male, 59 years)

‘There are not even enough physiotherapists to see to the urgent acute cases. How can anyone expect them to see to chronic conditions such as HIV/AIDS?’ (Participant 2, Male, 45 years)

#### Stigmatisation

Some of the respondents who expressed their opinion about the barriers they face in referring patients to physiotherapy mentioned stigmatisation as an obstacle to referral of patients. The following quotations illustrate participants’ sentiments:

‘Stigmatisation put some barriers in referring patients not only to physiotherapy but also to other departments of the hospital. Patients with HIV are afraid to expose themselves, fearing that they will be identified by the healthcare workers and stigmatised.’ (Participant 5, Male, 43 years)

‘Patients don’t want other people to know that they have the disease (HIV). They are afraid of being labelled.’ (Participant 6, Female, 39 years)

### Physicians’ recommendations for improved management

The majority of the participants made recommendations for improved management of patients with HIV-sensory neuropathy. The importance of a holistic approach should be emphasised by every person involved in patient care.

#### Multidisciplinary team

‘I think a multidisciplinary team would create more awareness of the role of each member of the team, including the physiotherapists … yeah … It is important in our setting … The management of the patients with HIV-sensory neuropathy depends on the attending physician, as we are the first line practitioner …’ (Participant 10, Male, 42 years)

‘I think we need to work as a team; actually, a multidisciplinary team would be of great significance in the management of these patients because it is the only chance to achieve the best treatment outcome for the patient.’ (Participant 4, Male, 59 years)

‘If we work together, for instance if the physiotherapist is around when I am assessing the patient, the physiotherapist can advise me to include physiotherapy as part of the treatment of the patient where there is a need … this is very important. The problem is that the physiotherapists are few compared to the number of patients ….’ (Participant 9, Male, 46 years)

#### Treatment guideline

Most of the participants reported that there is currently no treatment guideline for the management of patients with HIV-SN. According to literature, the recommended treatment for neuropathic pain includes general analgesics, antidepressants and changing or reducing the dosage of the offending HAART:

‘We do not have a treatment guideline for neuropathic pain associated with HIV at the moment; I often prescribe opioid analgesics, vitamins and tramadol along with antidepressants such as gabapentin and reduce the dose of the offending HAART drug.’ (Participant 10, Male, 42 years)

‘We do not have a guideline for the management of HIV-sensory neuropathy. Therefore the treatment depends upon the knowledge and experience of the attending physician. For me, I examine the underlying cause and if it is the HAART, I remove the drug, put the patient on a membrane stabiliser and prescribe vitamin B and analgesics.’ (Participant 5, Male, 43 years)

‘I think the guideline would help us to have more standardised and optimal treatment outcomes of the patients … and it will guide the physicians on the available treatment options including physiotherapy … of course we need that, but it is coming soon.’ (Participant 7, Female, 44 years)

‘We are now in preparation of the treatment guideline for the management of neuropathic pain in HIV/AIDS, including that of HIV-sensory neuropathy. I hope physiotherapy will be included as part of the management guideline for these patients.’ (Participant 10, Male, 42 years)

#### Increase physicians’ awareness

All of the participants agreed that awareness of physiciansregarding the role of physiotherapy in the management of patients with HIV-SN should be reiterated on several levels. The excerpts below are offered in support:

‘I think there is a need of increasing the physicians’ awareness of the role of physiotherapy in the management of medical cases like peripheral neuropathy because it is the knowledge that will facilitate the referral practice of the physician, of course if the physician does not understand the role of physiotherapy in these patients definitely will not refer the patients to them.’ (Participant 1, Female, 40 years)

‘Job training should include the education of both junior and senior doctors on the treatment options for patients with HIV-related sensory neuropathy. The specific role of every team member should be explicitly stated so that everyone knows what the other one does.’ (Participant 6, Female, 39 years)

The majority of the participants raised their concern at level of the awareness-raising on the part of the physiotherapy profession in general, and for their role in the management of patients with HIV-SN, as demonstrated below:

‘I think physiotherapists have not done enough to increase the awareness of their profession among other health professionals.’ (Participant 4, Male, 59 years)

‘Unlike medical professions such as medicine, pharmacy and nursing, physiotherapy is not well known in our country.’ (Participant 7, Female, 44 years)

One participant stated that physicians, as first line practitioners, should be aware of the role that each of the other healthcare professionals can play in patient care, including that of physiotherapists:

‘I cannot put all the blame on physiotherapists for not having created the awareness of their role in healthcare delivery. We physicians needed to be aware of the role of physiotherapy as we are the primary healthcare professionals responsible for referral to other healthcare professionals.’ (Participant 1, Female, 40 years)

## Discussion

An analysis of the major themes identified during the interviews revealed poor perceptions by physicians regarding the role of physiotherapy in the management of patients with HIV-SN. As people with HIV and AIDS are living longer, more chronic health problems such as peripheral neuropathy with its associated functional limitations manifests. Remarkably, there is a dearth of literature regarding the awareness of physiotherapy among medical doctors. Despite the scarcity of literature on this topic, research has shown negative attitudes towards the role of physiotherapy in healthcare delivery globally. Some of the physicians pointed out that physiotherapy management of patients with HIV-SN are less important compared to their own medical role. This could be the case for the initial diagnosis and treatment of the newly diagnosed patient with HIV-SN. However, physiotherapy might be more important in the later stages of the disease as it can assist with functional rehabilitation to increase the patients’ quality of life. Kruger (2010) highlighted the reality that physiotherapists play a subordinate role to the allopathic medical profession in most countries due to legislative, cultural and other challenges that prevent them from acting in full professional capacity of their knowledge and experience. In most countries physiotherapy is viewed as a paramedical occupation, a kind of optional supplementary to medical care (Amusat 2009), subordinate and auxiliary to allopathic medicine and surgery (Remennick & Shakhar 2003). Worthington *et al.* (2005) reported that physicians had negative perceptions of the role of rehabilitation professionals, including physiotherapists, in the management of HIV-related conditions. This negative attitude could hinder access to more integrated care for people living with HIV and AIDS (PLWHA) as physicians are the ‘gatekeepers’ to rehabilitation professionals in many developing countries. In addition, physicians perceived themselves as the major role players in the management of people living with HIV and AIDS (PLWHA), whereas rehabilitation professionals’ roles are less significant. The latter concur with Masyarakat (2005) and Solomon, Salvatori and Guenter (2003), who reported a lack of appreciation of the role of rehabilitation professionals in PLWHA, despite the prevalence of HIV-related disabilities that necessitate rehabilitation efforts.

Physicians’ negative perceptions and lack of awareness of what physiotherapists could offer in the rehabilitation of patients with HIV-SN were also recognised in their referral practices. The paucity of studies reporting on the trends of referral of patients with HIV-SN for physiotherapy intervention could be due to little attention being given to the subject. The negative perceptions not only result in the underutilisation of physiotherapy skills but also deprive patients’ of appropriate management (Masyarakat 2003). Fewer than a third of patients with HIV and AIDS diagnosed with functional limitations, impairments and participation restriction were referred for physiotherapy management (Kinirons & Do 2013). Since physiotherapists are legally obliged to follow prescribed treatment in many developing countries, it is of utmost importance that physicians should have a clear understanding of what physiotherapy can offer the affected patients. This coincides with findings from a study by Sherer *et al.* (2002), who validate the multidisciplinary team model of HIV care, and suggest that health services that are tailored to the needs of patients lead to better care and improved health outcomes.

The findings of the present study indicate that the majority of the physicians at the participating hospital have inadequate knowledge of the role physiotherapy can play in the management of patients with HIV-SN, and are therefore not referring their patients. No studies were found regarding physicians’ knowledge of the role of physiotherapy in the management of HIV-SN. However, several studies reported on physicians’ limited knowledge of the role of rehabilitation professionals in PLWHA in the era of HAART (Kinirons & Do 2013; Worthington *et al.* 2005). These findings also align with a participant’s response in a study conducted by Chetty and Maharaj (2013):

‘I don’t think I know exactly what the physiotherapist can do. I mean relaxation and other things I heard from today’s discussion. I thought they can massage and do exercise and walk the patients, but if we can learn more about them, that can help us work together.’ (p. 173)

Limited knowledge of the role of physiotherapy in healthcare delivery was also mentioned by several researchers (Dunkel 2004; Jackson 2004).

In addition to physicians’ inadequate knowledge and poor perceptions of the role of physiotherapy in the management of patients with HIV-SN, the perception of patients’ possible negative attitudes towards physiotherapy professionals, as experienced by the physicians, was also identified as one of the reasons for low referral rates. Physicians reported that patients may believe the physiotherapists would hurt them. Crout, Tweedie and Miller (1998) made a similar observation and suggested the need to educate patients and increase their awareness regarding physiotherapy. These findings indicate that it is imperative for physiotherapists to recognise and respond to feedback from service users. In addition, physiotherapists need to create an awareness of their role in healthcare delivery in order to improve public perceptions of them and their service.

Stigmatisation is a major concern for PLWHA. Studies have documented HIV and AIDS-related stigmatisation in contexts such as the community, the family, the workplace as well as the healthcare system (Bharat & Aggleton 1999; Bharat, Singhanetra-Renard & Aggleton 1998). In addition, negative attitudes from healthcare staff also generated anxiety and fear among PLWHA. There is an urgent need to extend awareness among healthcare staff concerning their legal duties and responsibilities towards patients with HIV and AIDS in particular. It is not enough to raise awareness about HIV and AIDS and its transmission routes for instance. Enabling environments should be created to facilitate the formation of support groups so that stigmatisation is challenged. What is urgently needed is a policy supported by a law that will ensure the protection of PLWHAs’ rights (Bharat, Aggleton & Tyrer 2001).

Inadequate access to rehabilitation services is a significant problem in many developing countries (Amusat 2009; Mji *et al.* 2009). This is in line with another concerning finding from the present study, because the low number of physiotherapists employed at the hospital could hinder access to physiotherapy. Access to rehabilitation professionals, including physiotherapists, was among the barriers to access to rehabilitation services for PLWHA (Worthington *et al.* 2008). Increasing accessibility considerably facilitated physicians’ rates of referrals (Jørgensen & Olesen 2001; Masyarakat 2005).

### Study limitations

This study presents the views of 10 physicians who volunteered to participate. Their views may differ from physicians working at other hospitals and in different countries. The results are therefore not generalisable to physicians in general. Nor is it possible to determine whether the issues that emerged in the study represent general features of the role of physiotherapy in the healthcare delivery system. Replication of this work in other contexts may clarify this issue.

## Conclusion

In some African countries referral for physiotherapy is still dependent on medical doctors. This clearly indicates that an awareness of the rehabilitatory role of physiotherapy among medical doctors is very important when it comes to the referral of patients. Inter-professional education is imperative for minimising the stereotypes that may exist across professions, thereby improving awareness of specific roles in patient management. This could improve knowledge of the role of other professionals in the management and rehabilitation of affected patients and consequently improve perceptions and facilitate referrals of patients with HIV-SN for more integrated care.
